# Effects of Magnesium Orotate, Benfotiamine and a Combination of Vitamins on Mitochondrial and Cholinergic Function in the TgF344-AD Rat Model of Alzheimer’s Disease

**DOI:** 10.3390/ph14121218

**Published:** 2021-11-24

**Authors:** Christian Viel, Adrian T. Brandtner, Alexander Weißhaar, Alina Lehto, Marius Fuchs, Jochen Klein

**Affiliations:** 1 Institute of Pharmacology and Clinical Pharmacy, College of Pharmacy, Goethe University, Max-von-Laue-Str. 9, 60438 Frankfurt am Main, Germany; viel@em.uni-frankfurt.de (C.V.); adrian.brandtner@ukbonn.de (A.T.B.); alexander.weisshaar@oc.uni-stuttgart.de (A.W.); lehto@em.uni-frankfurt.de (A.L.); m.fuchs@em.uni-frankfurt.de (M.F.); 2 Institute of Physiology I, Rheinische Friedrich-Wilhelms-Universität, Sigmund-Freud-Straße 25, Venusberg-Campus 1, 53105 Bonn, Germany; 3 Institute of Organic Chemistry, University of Stuttgart, Pfaffenwaldring 55, 70569 Stuttgart, Germany

**Keywords:** acetylcholine, Alzheimer’s disease, microdialysis, glucose, lactate, mitochondrial respiration, complex I, electron transfer system, TgF344-AD, hippocampus

## Abstract

Glucose hypometabolism, mitochondrial dysfunction, and cholinergic deficits have been reported in early stages of Alzheimer’s disease (AD). Here, we examine these parameters in TgF344-AD rats, an Alzheimer model that carries amyloid precursor protein and presenilin-1 mutations, and of wild type F344 rats. In mitochondria isolated from rat hippocampi, we found reductions of complex I and oxidative phosphorylation in transgenic rats. Further impairments, also of complex II, were observed in aged (wild-type and transgenic) rats. Treatment with a “cocktail” containing magnesium orotate, benfotiamine, folic acid, cyanocobalamin, and cholecalciferol did not affect mitochondrial activities in wild-type rats but restored diminished activities in transgenic rats to wild-type levels. Glucose, lactate, and pyruvate levels were unchanged by age, genetic background, or treatment. Using microdialysis, we also investigated extracellular concentrations of acetylcholine that were strongly reduced in transgenic animals. Again, ACh levels in wild-type rats did not change upon treatment with nutrients, whereas the cocktail increased hippocampal acetylcholine levels under physiological stimulation. We conclude that TgF344-AD rats display a distinct mitochondrial and cholinergic dysfunction not unlike the findings in patients suffering from AD. This dysfunction can be partially corrected by the application of the “cocktail” which is particularly active in aged rats. We suggest that the TgF344-AD rat is a promising model to further investigate mitochondrial and cholinergic dysfunction and potential treatment approaches for AD.

## 1. Introduction

The rising prevalence of Alzheimer’s disease (AD) is a substantial burden to individuals and to health care systems, making the development of new therapeutic concepts for treating AD an important goal [1,2]. Currently available drugs, such as acetylcholinesterase (AChE) inhibitors and memantine, merely act symptomatically and only delay the progression of the disease. Unfortunately, disease-modifying drugs targeting, for instance, the reduction of Aβ peptides did not achieve clinical endpoints [3]. Henceforth, recent suggestions for AD drug developments focused on other aspects of the disease, especially energy metabolism and mitochondrial function [4]. The reduction of cerebral glucose consumption is a very early marker of AD, and reductions of glycolytic enzymes and enzymes of the tricarboxylic acid cycle were described in AD brains decades ago [5,6]. Several papers also described reductions of mitochondrial complex activities in AD patients [7,8]. Transgenic mouse models of AD also display signs of mitochondrial dysfunction [9,10,11]. The deficits of brain energy metabolism led to the idea of “brain energy rescue” as a promising therapeutic approach for AD [4].

In the present study, we investigated a transgenic rat model of AD, the TgF344-AD rat [12]. The animals express mutant human amyloid precursor protein (APPsw) and presenilin-1 (PS1ΔE9) genes and exhibit Aβ plaque formation, hyperphosphorylation and aggregation of intracellular tau proteins, neurophysiological and behavioral abnormalities and signs of neurodegeneration that reflect AD patients’ pathology [12,13]. Mitochondrial complexes and acetylcholine levels have not been investigated yet. In our study, we used animals at the age of 6–7 months when Aβ peptide formation is increased 2–3 fold and presenilin-1 concentration is up to six times higher, but cognition is normal, and at 15–16 months when the rats show clear signs of cognitive impairment. We found impairments of mitochondrial and cholinergic function which were particularly prominent in aged rats.

Following the observation of mitochondrial and cholinergic deficits, we tested whether a treatment with magnesium orotate and/or with certain vitamins (benfotiamine, folic acid, vitamin B12 and D3) can increase mitochondrial oxygen consumption and acetylcholine levels. With a cocktail of these nutrients (magnesium orotate 500 mg/kg, benfotiamine 300 mg/kg, folic acid 10 mg/kg, vitamin B12 1 mg/kg and vitamin D3 5 µg/kg), we found impressive improvements of mitochondrial and cholinergic function in this rat AD model. Our data support previous clinical findings that a treatment with a mixture of nutrients may have a beneficial influence on brain energy metabolism and neuronal function in AD.

## 2. Results

**Figure 1:**
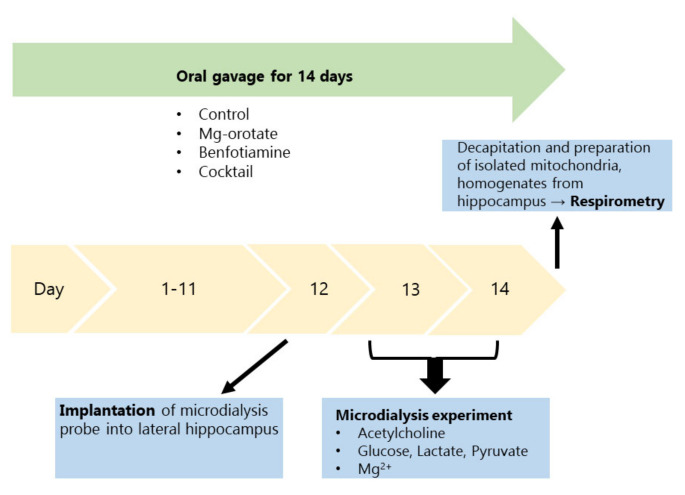
Flow chart of the experimental design. Rats received either nutrients or vehicle by oral gavage for 14 days. The microdialysis probe was implanted on day 12. Microdialysis experiments (open field, scopolamine perfusion) were performed on day 13 and 14 to collect samples for acetylcholine, energy metabolites and magnesium measurements. Respirometry was done on day 14 with freshly prepared mitochondria from hippocampi of rats. Homogenates for AChE and ChAT measurements were also prepared on day 14.

The time line of the experiments is shown in Figure 1.

### 2.1. Mitochondrial Respiration

**Figure 2:**
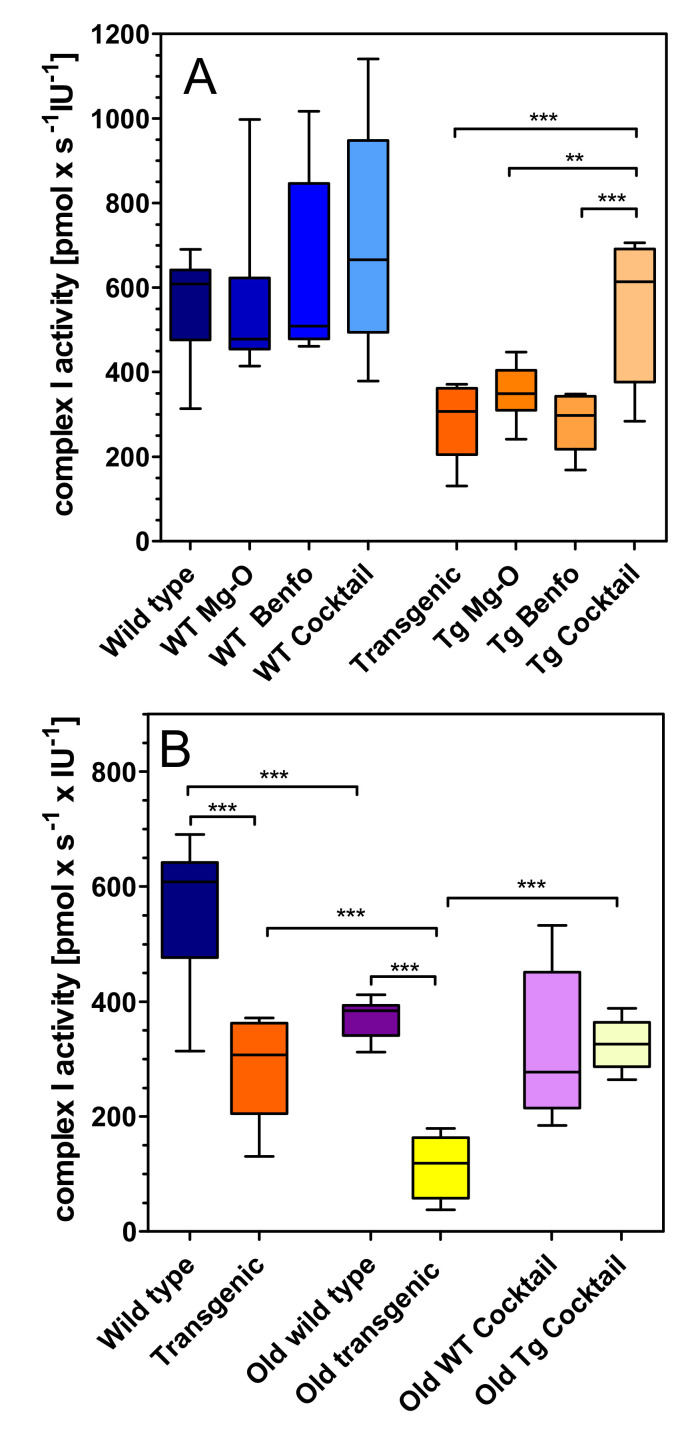
Oxygen flux of complex I in (**A**) young wild-type and transgenic rats and (**B**) young and old rats treated with cocktail. Substrates, uncoupler and inhibitors were used to measure complex I as described in methods. Raw data were normalised to mitochondrial citrate synthase activity. A: (left) Treatment in wild type (WT), (right) treatment in transgenic (Tg) animals. Statistical analysis: Data are box plots (lower and upper quartile) with whiskers (*n* = 8–10) and were analysed by one-way ANOVA followed by Bonferroni’s multiple comparison test: (a) F3,34 = 0.96, *p* = 0.43; (b) F3,31 = 11.78, *p* < 0.001. ** *p* < 0.01, *** *p* < 0.001 B: Genotype, treatment and age related effects. Statistical analysis: Data are box plots (lower and upper quartile) with whiskers (*n* = 8–10) and were analysed by unpaired *t*-test. *** *p* < 0.001.

Mitochondrial complexes were measured ex vivo in isolated hippocampal mitochondria. Compared to wild-type rats, young transgenic animals (at 6–7 months of age) had reduced complex I respiration (−50%; Figure 2A). While magnesium orotate and benfotiamine did not significantly affect respiration, the administration of the cocktail caused an increase of complex I respiration in transgenic rats (Figure 2A). In aged rats (15–16 months of age), we observed a reduction of complex I respiration both in wild-type (−34%) and in transgenic rats (−60%) (Figure 2B). The treatment with cocktail was very effective in transgenic rats: it increased respiration by 60% so that complex I respiration in transgenic rats reached the activity of wild-type animals (Figure 2B).

**Figure 3:**
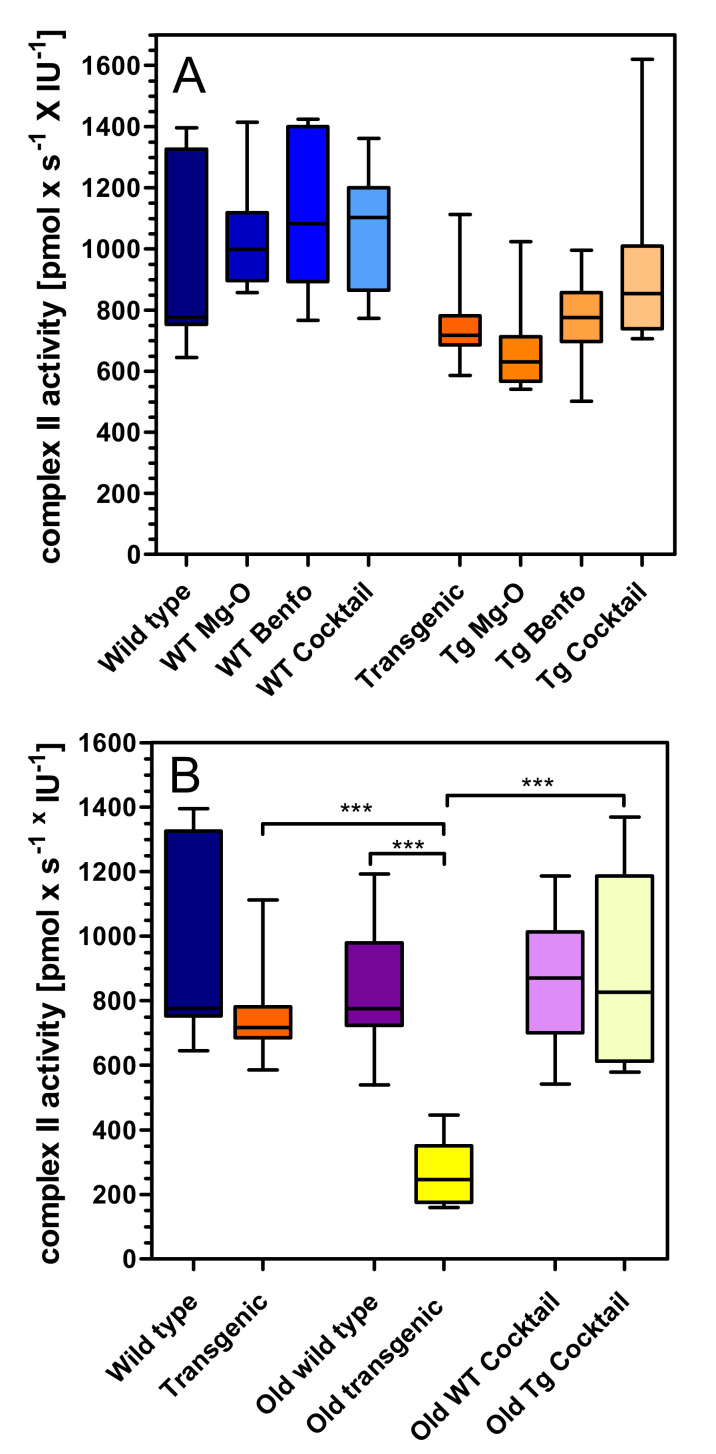
Oxygen flux of complex II in (**A**) young wild-type and transgenic rats and (**B**) young and old rats treated with cocktail. Substrates, uncoupler and inhibitors were used to measure complex II as described in methods. Raw data were normalised to mitochondrial citrate synthase activity. A: (left) Treatment in wild type (WT), (right) treatment in transgenic (Tg) animals. Statistical analysis: Data are box plots (lower and upper quartile) with whiskers (*n* = 7–10) and were analysed by one-way ANOVA followed by Bonferroni’s multiple comparison test: (a) F3,36 = 0.99, *p* = 0.41; (b) F3,33 = 2.44, *p* = 0.08. B: Genotype, treatment and age related effects. Statistical analysis: Data are box plots (lower and upper quartile) with whiskers (*n* = 7–10) and were analysed by unpaired *t*-test. *** *p* < 0.001.

Complex II respiration was slightly (−19%) but not significantly reduced in young transgenic rats compared to wild-type rats (Figure 3A), and the treatments were inactive in young rats. In old transgenic rats, complex II respiration was significantly reduced when compared to old wild-type rats (−68%, *p* < 0.01) (Figure 3B). Again, cocktail treatment was inactive in wild-type rats but restored complex II respiration deficits in old transgenic animals to its full extent (+70%) (Figure 3B).

**Figure 4:**
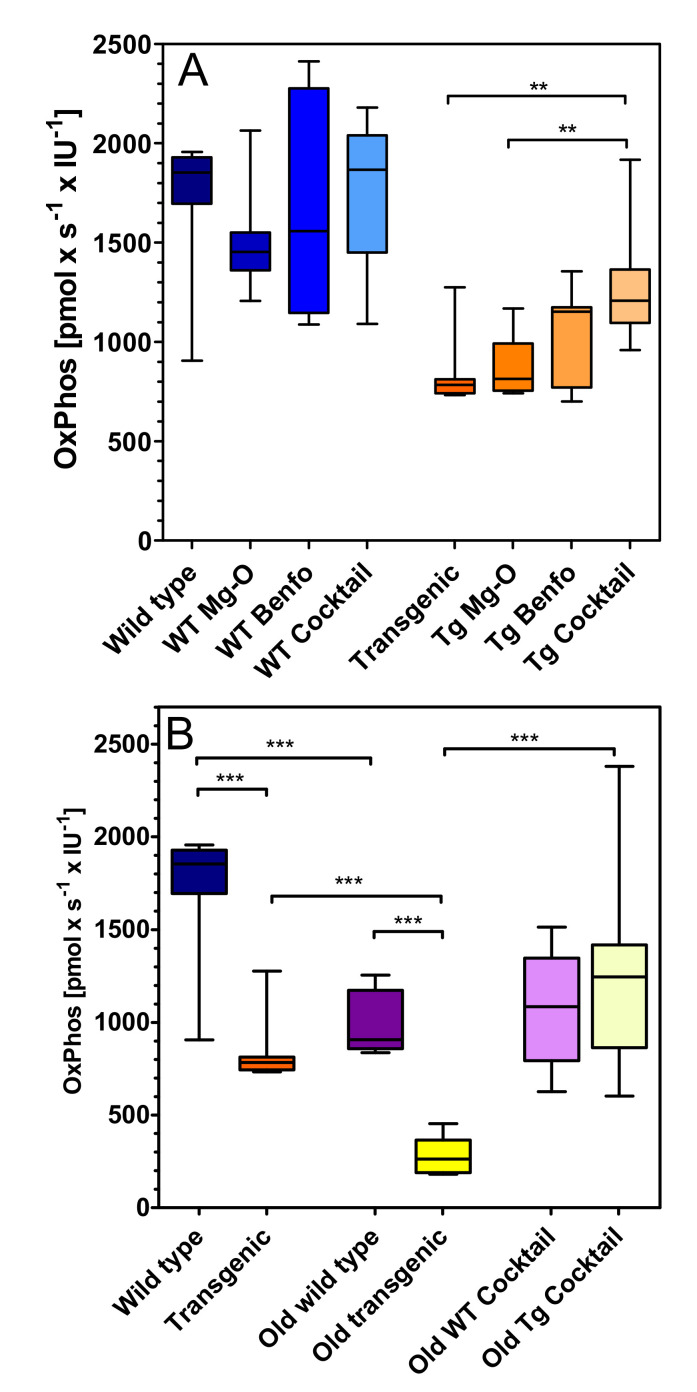
Oxygen flux related to oxidative phosphorylation (OxPhos) in (**A**) young wild-type and transgenic rats and (**B**) young and old rats treated with cocktail. Substrates, uncoupler and inhibitors were used to measure oxidative phosphorylation-related oxygen consumption as described in methods. Raw data were normalised to mitochondrial citrate synthase activity. A: (left) Treatment in wild type (WT), (right) treatment in transgenic (Tg) animals. Statistical analysis: Data are box plots (lower and upper quartile) with whiskers (*n* = 7–10) and were analysed by one-way ANOVA followed by Bonferroni’s multiple comparison test: (a) F3,33 = 0.84, *p* = 0.48; (b) F3,33 = 7.06, *p* = 0.001. ** *p* < 0.01 B: Genotype, treatment and age related effects. Statistical analysis: Data are box plots (lower and upper quartile) with whiskers (*n* = 8–10) and were analysed by unpaired *t*-test. *** *p* < 0.001.

In line with the findings listed above, OxPhos capacity was significantly lower in young transgenic rats compared to wild-type rats (−51%, *p* < 0.01) (Figure 4). Cocktail treatment significantly improved OxPhos respiration by 34% in young transgenic rats (*p* < 0.01; Figure 4A) but not in young wild-type animals. The age-related decrease of OxPhos capacity was 43% in wild-type and 66% in transgenic rats (*p* < 0.01; Figure 4B). In old rats, OxPhos respiration was 71% lower in transgenic than in wild-type rats (*p* < 0.01). Cocktail treatment was inactive in old wild-type animals but completely restored OxPhos respiration in old transgenic animals (+78%; *p* < 0.01).

**Figure 5:**
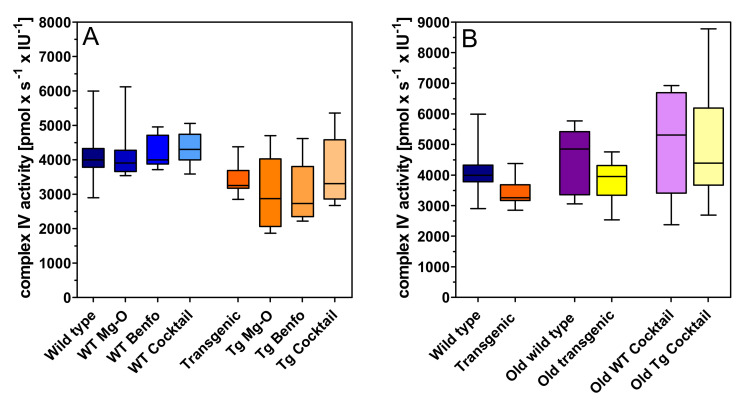
Oxygen flux of complex IV in (**A**) young wild-type and transgenic rats and (**B**) young and old rats treated with cocktail. Substrates, uncoupler and inhibitors were used to measure oxidative phosphorylation related oxygen consumption as described in methods. Raw data were normalised to mitochondrial citrate synthase activity. A: (left) Treatment in wild type (WT), (right) treatment in transgenic (Tg) animals. Statistical analysis: Data are box plots (lower and upper quartile) with whiskers (*n* = 8–10) and were analysed by one-way ANOVA followed by Bonferroni’s multiple comparison test: (a) F3,37 = 0.19, *p* = 0.90; (b) F3,34 = 1.05, *p* = 0.38. B: Genotype, treatment and age related effects. Statistical analysis: Data are box plots (lower and upper quartile) with whiskers (*n* = 8–10) and were analysed by unpaired *t*-test.

Complex IV respiration was unchanged in young and old transgenic animals compared to wild-type rats (Figure 5). None of the treatments had significant effects in either rat strain (Figure 5).

### 2.2. Energy Metabolites

We used the microdialysis technique to monitor energy metabolism in the brain. The extracellular concentrations of glucose and lactate were 157 ± 16 µM and 183 ± 19 µM, respectively (not corrected for recovery). Basal extracellular glucose, lactate and pyruvate levels were not influenced by age or transgenes (Table 1). Moreover, the treatments did not significantly affect energy metabolites in young or old rats.

### 2.3. Extracellular Magnesium Levels

**Figure 6:**
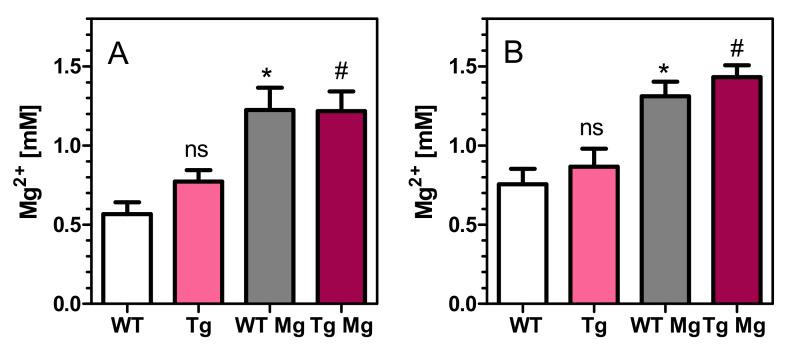
Extracellular concentrations of magnesium (not corrected for recovery) as determined by microdialysis. (**A**) Young (6–7 month old) rats. (**B**) Old (15–16 month old) rats. Magnesium levels were measured in untreated wild-type and transgenic rats (WT, Tg) and after treatment with magnesium orotate (WT Mg, Tg Mg). Data are means ± SEM (*n* = 4–19) and were analysed by one-way ANOVA followed by Bonferroni’s multiple comparison test: (**A**) F_3,46_ = 5.82, *p* < 0.01. (**B**) Old rats: F_3,46_ = 11.71, *p* < 0.001. ns, not significant vs. WT. * *p* < 0.05 vs. WT rats. # *p* < 0.05 vs. Tg rats.

Samples for the determination of extracellular magnesium concentrations were obtained on day 14 of the microdialysis experiments (Figure 6). Samples were taken one hour after oral gavage of magnesium orotate. In young rats, transgenic rats had slightly higher magnesium levels than wild-type rats (0.77 vs. 0.57 mM on average), but the difference did not reach significance (*p* > 0.05) and largely disappeared in old rats (0.87 vs. 0.76 mM, *p* = 0.50). All animal groups (young vs. old, wild-type vs. transgenic) responded with an increase of extracellular magnesium in the brain (Figure 6). Taken together, all untreated animals displayed average magnesium levels of 0.69 mM whereas animals treated for 14 days with magnesium orotate reached average levels of 1.22 mM (Figure 6). This difference of extracellular magnesium levels was highly significant (*p* < 0.01).

### 2.4. Acetylcholine Levels

**Figure 7:**
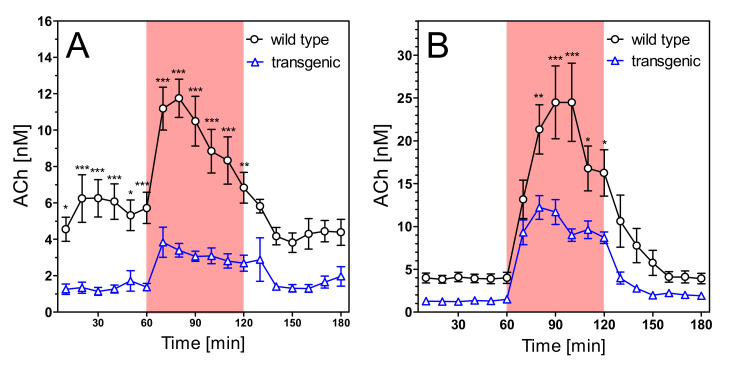
Extracellular ACh concentrations in hippocampus of 6–7 months old wild-type and transgenic rats during open field test (**A**) and under scopolamine perfusion (**B**). The red area represents the intervention (open field exposure or scopolamine perfusion) from 60–120 min. Data are means ± SEM (*n* = 7) and were analysed by two-way ANOVA for genotype as variable followed by Bonferroni’s multiple comparison test: (**A**) F1,204 = 6.32, *p* < 0.001. (**B**) F1,204 = 4.6, *p* < 0.01. * *p* < 0.05, ** *p* < 0.01, *** *p* < 0.001.

**Figure 8:**
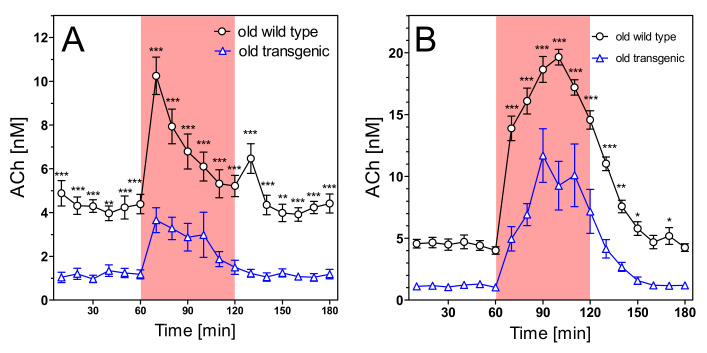
Extracellular ACh concentrations in hippocampus of 15–16 months old wild-type and transgenic rats during open field test (**A**) and under scopolamine perfusion (**B**). The red area represents the intervention (open field exposure or scopolamine perfusion) from 60–120 min, Data are means ± SEM (*n* = 8–9) and were analysed by two-way ANOVA for genotype as variable followed by Bonferroni’s multiple comparison test: (**A**) F1,255 = 4.23, *p* < 0.001 (**B**) F1,255 = 5.29, *p* < 0.001. * *p* < 0.05, ** *p* < 0.01, *** *p* < 0.001.

Extracellular concentrations of acetylcholine (ACh) were 5.70 ± 0.75 nM in young wild-type rats and 1.35 ± 0.22 nM in young transgenic rats (Figure 7A; not corrected for recovery). The difference is highly significant (*n* = 7 each; *p* < 0.001). Aging (Figure 8A) reduced the ACh levels slightly but not significantly. However, the old wild-type rats still had much higher levels of hippocampal ACh (4.35 ± 0.33 nM) than old transgenic rats (1.16 ± 0.18 nM) (*n* = 8–9, *p* < 0.001).

In spite of the strong differences in basal ACh levels, both strains responded similarly to stimulatory conditions. Exposure to the open field increased ACh release by 2–3 fold in both strains, in young and old animals (Figure 7A and Figure 8A). Similarly, infusion of scopolamine increased ACh release 5–6-fold in all groups (Figure 7B and Figure 8B). In absolute terms, maximum concentrations of ACh were significantly higher in wild-type rats (Figure 7 and Figure 8), but in relative terms (percentage vs. basal levels) the responses were not significantly different (data not illustrated).

**Figure 9:**
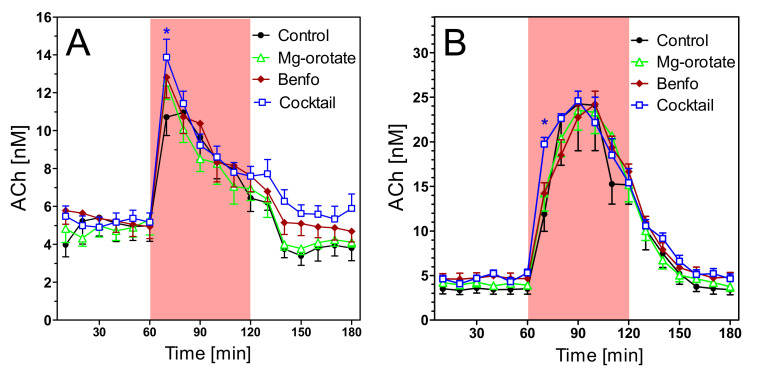
Extracellular ACh concentrations in hippocampus of young (6–7 months old) wild-type rats during during open field test (**A**) and under scopolamine perfusion (**B**). The rats were submitted to four different treatments for 14 days: no treatment (“control”), magnesium orotate, benfotiamine and cocktail. The red area represents the intervention (open field exposure or scopolamine perfusion) from 60–120 min. Data are means ± SEM (*n* = 8–9) and were analysed by two-way ANOVA for treatment as variable followed by Bonferroni’s multiple comparison test: (**A**) F3,527 = 1.02, *p* = 0.47, * *p* < 0.05. (**B**) F3,527 = 0.77, *p* = 0.78. * *p* < 0.05.

**Figure 10:**
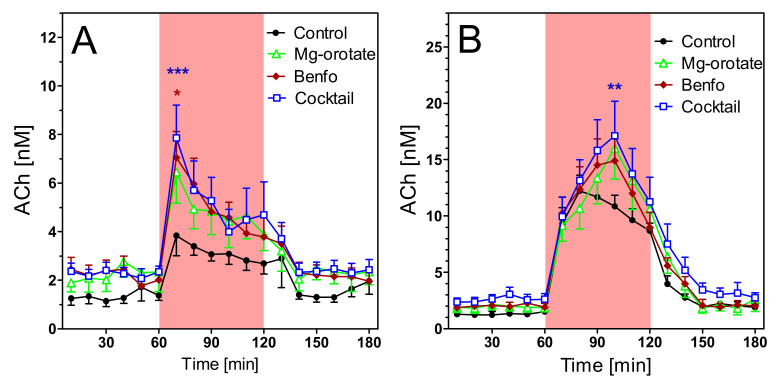
Extracellular ACh concentrations in hippocampus of young (6–7 months old) transgenic rats during open field test (**A**) and during scopolamine perfusion (**B**). The rats submitted to 4 different treatments for 14 days: no treatment (“control”), magnesium orotate, benfotiamine and cocktail. Red area represents intervention (open field exposure or scopolamine perfusion) from 60–120 min. Data are means ± SEM (*n* =7–8) and were analysed by two-way ANOVA for treatment as variable followed by Bonferroni’s multiple comparison test: (**A**) F3,442 = 0.90, *p* = 0.27 (**B**) F3,442 = 1.04, *p* = 0.41. Comparison of “control” to “cocktail”: F1,204 = 4.52; *p* = 0.055. * *p* < 0.05, ** *p* < 0.01, *** *p* < 0.001.

In young wild-type rats, the treatments with nutrients did not affect the time course of ACh release after open field or scopolamine, as shown in Figure 9. Only the initial increase 15 min past stimulation was significantly higher in the cocktail-treated animals. In young transgenic rats in the open field, however, ACh release was higher after treatment with either benfotiamine, magnesium orotate, or cocktail (Figure 10A). Interestingly, the treatment effect was small when monitored during scopolamine infusion (Figure 10B) although the absolute amount of released ACh was much higher under this condition.

**Figure 11:**
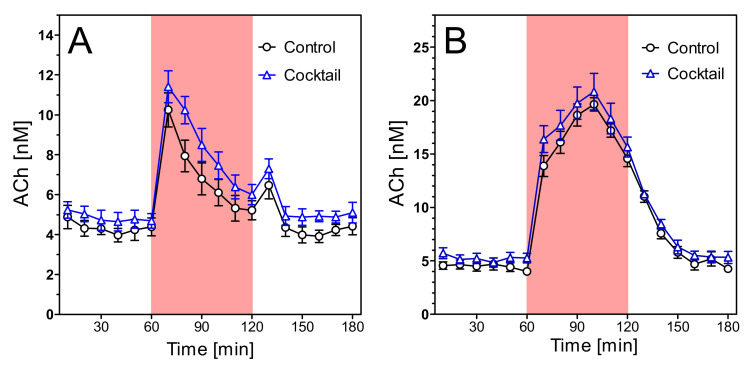
Extracellular ACh concentrations in hippocampus of 15–16 months old wild-type rats during open field test (**A**) and during scopolamine perfusion (**B**). The rats were submitted to two different treatments: no treatment (“control”) and cocktail. The red area represents the intervention (open field exposure) from 60–120 min. Data are means ± SEM (*n* = 9–10) and were analysed by two-way ANOVA for treatment as variable followed by Bonferroni’s multiple comparison test: (**A**) F1,289 = 2.24, *p* = 0.15. (**B**) F1,289 = 1.93, *p* = 0.18.

**Figure 12:**
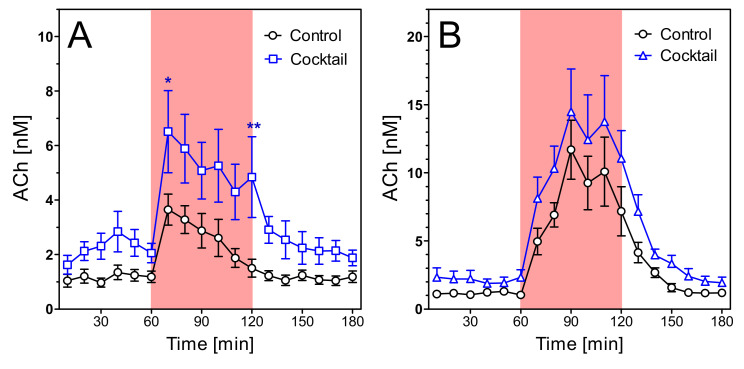
Extracellular ACh concentrations in hippocampus of 15–16 months old transgenic rats during open field test (**A**) and during scopolamine perfusion (**B**). The rats were submitted to two different treatments: no treatment (“control”) and cocktail. The red area represents the intervention (open field exposure or scopolamine perfusion) from 60–120 min. Data are means ± SEM (*n* = 8) and were analysed by two-way ANOVA for treatment as variable followed by Bonferroni’s multiple comparison test: (**A**) F1,238 = 4.90, *p* = 0.04. (**B**) F1,238 = 2.47, *p* = 0.14. *, *p* < 0.05; **, *p* < 0.01.

The results with old rats corroborated the findings with young rats. Old wild-type animals did not respond to the treatment with cocktail with a significantly altered ACh release (Figure 11). In old transgenic rats, however, ACh levels during open field exposure were significantly increased, whereas no significant effect was registered during scopolamine infusion (Figure 12).

### 2.5. Cholinergic Enzymes

**Figure 13:**
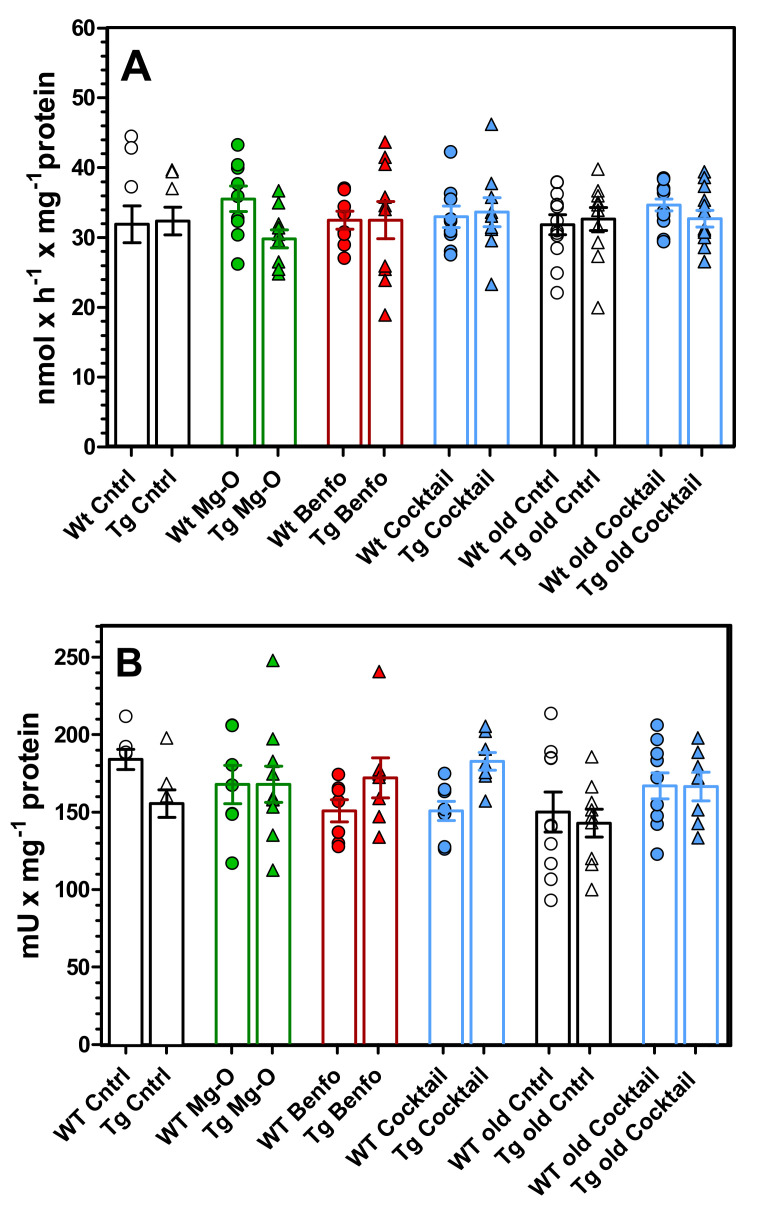
Enzyme activities of (**A**) choline acetyltransferase (ChAT) and (**B**) acetylcholinesterase (AChE) normalised to protein content. Data are means ± SEM (*n* = 7–9) and were analysed by one-way ANOVA. (**A**) ChAT: F11,120 = 0.71, *p* = 0.73; (**B**) AChE: F11,96 = 1.76, *p* = 0.07.

As shown in Figure 13, the activity of choline acetyltransferase (ChAT) in hippocampal homogenates were 34.2 ± 3.32 nmol/h mg·protein and of acetylcholinesterase 184 ± 6.45 mU/mg protein. These activities were not significantly changed by age, transgene, or any of the treatments.

## 3. Discussion

Mitochondrial dysfunction as a pathological feature of AD was described in numerous studies (see Introduction). More recently, changes of gene expression were documented in patients with AD or MCI, a preliminary stage of AD. These changes concerned nuclear- as well as mitochondria-encoded OxPhos genes involved in ATP production [14,15]. Mitochondrial fragmentation resulting in dysfunctional mitochondria was observed in hippocampal neurons of AD patients [16]. This resulted in ATP depletion and stimulation of the AMP-activated protein kinase. A drastic reduction of mitochondria in presynaptic terminals of cortical areas and abnormal mitochondrial morphology were described in [17]. Several studies described complex I activities as particularly vulnerable in AD patients, e.g., by proteomics [18]. An increase in reactive oxygen production (ROS) was reported [19] as well as an increase of 8-oxoguanine, a byproduct of increased oxidative stress [20]. With respect to mitochondrial distribution within cells, evidence for perturbed axonal transport was found in pyramidal neurons of AD brains. Markers of mitochondrial fusion (DLP1, OPA, Mfn1, Mfn2) were significantly reduced, whereas a marker of mitochondrial fission (Fis1) was significantly increased, leading to a reduced density of mitochondria in neurons [21]. Of note, similar changes were found in mouse models of AD, including changes of mitochondrial transport, reduced ATP levels, and increased expression of the mitochondrial fission protein Fis1 [22,23,24]. In an APP/PS1 mouse model of AD, the mtDNA/nDNA ratio was decreased in cortex and hippocampus [25].

The many reports of mitochondrial dysfunction gave the rationale for our present study in which we first focused on mitochondrial respiration in the Tg-F344 AD rat model. Arguably, rats may be preferable to mice because they are ten times larger than mice and physiologically closer to humans. Their metabolic rate is not quite as high as in the mouse, and in fact, rats were the major model for neurochemistry before transgenic mice were available. In the present study, we found significantly lower activities for complex I of the electron transport chain in transgenic rats, and reduced OxPhos capacity at the age of six months when cognitive function is not yet affected. Minor reductions were observed for complex II and complex IV. Single administrations of benfotiamine did not significantly affect mitochondrial respiration, neither did magnesium orotate, although magnesium levels in the brain extracellular fluid increased. The combination of the two compounds with cholecalciferol, folic acid, and vitamin B12 (“the cocktail”), however, significantly increased mitochondrial respiration in young transgenic rats, especially with respect to complex I and OxPhos activities.

The remarkable effectiveness of the “cocktail” in transgenic rats was corroborated by the data obtained in aged animals. At 15 months, wild-type F344 rats had reduced mitochondrial respiration in isolated mitochondria from hippocampus. While complexes II and IV activities were slightly reduced, complex I and OxPhos activities were significantly reduced by approximately 50% (*p* < 0.01). The changes in transgenic rats were even more impressive: While their complex I and OxPhos activities were already halved at the age of six months, they decreased further with aging and reached less than 20% of young wild-type controls at 15 months of age. It is not known how this severe reduction of mitochondrial respiration comes about. Some hints from mouse models are the previously described structural changes in mitochondrial morphology and changed gene expression patterns (see above). Impaired mitochondrial respiration of hippocampal and/or synaptic mitochondria and reduced expression of complex I, III, and IV subunits together with reduced complex IV activities were also reported in murine amyloid models [26,27] but none of these studies clearly identified the link between amyloid disposition and mitochondrial dysfunction. A possible explanation may be direct interactions between intracellular amyloid and the mitochondrial membrane [28].

We did not find any significant change of mitochondrial complex IV under any condition, and this finding requires a comment. In our study, complex IV respiration was measured ex vivo under non-physiological conditions. In isolated hippocampal mitochondria, maximum complex IV activity was measured after adding the electron donor TMPD (N,N,N′,N′-tetramethyl-*p*-phenylenediamine) and ascorbate to regenerate it. Thus, the conditions allowed maximum oxygen consumption by complex IV which yielded similar values for all conditions. Our measurements of complex IV, therefore, do not contradict the reduction of OxPhos activities, and they do not exclude potential limitations of complex IV activities in vivo. However, they are at variance with studies in mice in which reduced expression of complex IV subunits were reported.

Another possible explanation of reduced mitochondrial respiration in aging and transgenic rats would be lack of energy metabolites. Reductions of glucose or impaired functioning of the citric acid cycle would easily explain reduced electron transport capacity in vivo. To test substrate availability and possible impairment of the citric acid cycle, we monitored energy metabolites by microdialysis. In no case did we find any change of energy metabolites in our animal model. We conclude from these findings that the availability of energy substrates did not cause any of the changes observed in aging and/or transgenic rats.

The individual activities of complexes I and II reflect ex vivo activities in the presence of abundant substrate, and the OxPhos capacity is probably the best surrogate parameter to judge on the quality of mitochondrial respiration because it reflects mitochondrial oxygen consumption under optimal substrate concentrations for complex I and II and abundant ADP supply. Complex I and OxPhos were reduced by both aging and transgenic expression of amyloid, whereas complex II was significantly reduced only in transgenic rats at 15 months of age. In old transgenic rats, we observed the most striking finding of our study, namely the near normalization of mitochondrial respiration by a two-week oral treatment with the “cocktail”. This treatment with the cocktail increased oxygen consumption by more than three-fold so that respiration in cocktail-treated transgenic rats equaled the respiration in healthy aged controls. This impressive effect was unexpected and may have translational value (see below).

The mechanism of the “cocktail effect” is elusive at present. It must be noted, however, that aged wild-type rats did not profit from cocktail treatment which means that the treatment improved impairments of mitochondrial respiration that were induced by the expression of the mutant APP and PS-1 genes. It seems most likely that intracellular amyloid formation caused an impairment of electron flux through the complexes I and II. With respect to the molecular mechanism of action, several possibilities can be considered. A deficiency of cofactors (NADH or FADH2) seems less likely because neither nicotinamide nor riboflavin were part of the cocktail treatment. Thiamine (vitamin B1) was given as its precursor, benfotiamine. Both α-ketoglutarate dehydrogenase and pyruvate dehydrogenase use thiamine pyrophosphate as a cofactor, and both enzymes were reported to be reduced in Alzheimer brains [29]. Hence, befotiamine may have contributed to the activity of the cocktail. AD patients evidently have low blood concentrations of vitamin B1 and reduced B1-dependent enzyme activities [30,31]. However, the single treatment with benfotiamine was not effective, at least not in young transgenic rats. The results with magnesium orotate were comparable to those with benfotiamine. Neither caused a significant effect in young transgenic rats, but they may have contributed to the effects of the cocktail. We show here that the extracellular magnesium level in the brain increased by an average of 43% within two weeks of magnesium orotate administration. Although our knowledge on the dynamics of magnesium in the CNS remains incomplete [32], orally given magnesium clearly enters the brain and causes increases of its extracellular concentration which may have secondary effects, either in increasing intracellular magnesium and/or in partially reducing calcium conductances, e.g., through NMDA receptors. Intracellularly, magnesium is an important binding partner for ATP and a cofactor for numerous enzymes. Of note, magnesium threonate treatment had positive effects in the APPSWE/PS1ΔE9 mouse model. In this study, BACE activity and NMDA receptor expression in hippocampal tissue were normalized by treatment with magnesium threonate [33].

Reports on the central effects of orotic acid are also fragmentary [34]. Orotic acid is an intermediate of pyrimidine biosynthesis. AD patients have altered mRNA levels of genes involved in pyrimidine synthesis [35]. Dihydroorotate dehydrogenase is located on the inner mitochondrial membrane and can serve as an electron donor to the electron transfer system (ETS) connecting pyrimidine synthesis to OxPhos. An increase of pyrimidines which serve DNA and RNA synthesis in neurons may also contribute to the effects of magnesium orotate.

Our study did not test individual constituents of the cocktail in old rats. However, it may be safely presumed that several constituents of the cocktail are required for full activity. Folic acid and vitamin B12, both parts of the cocktail, are methyl donors and acceptors within the C1-metabolism. They are relevant for pyrimidine and purine synthesis, but may also be involved in methylations, e.g., of histones and may influence gene expression from the nuclear genome. High homocysteine levels, a hallmark of reduced C1-metabolism, was described in Alzheimer patients and linked to neurodegeneration [36]. Vitamin D, the fifth part of the cocktail, activates nuclear receptors which are present in almost all cell types including neurons. A synergism of the five ingredients is easily conceivable but requires further studies to be elucidated.

In light of the impressive effects of the cocktail treatment, the question arose as to the consequences of these effects for neuronal transmission. We chose to measure acetylcholine (ACh) levels to monitor neurotransmission on the hippocampus. ACh is a useful indicator of neuronal transmission in this study for several reasons. For instance, it is an exclusively neuronal product, and the synthesis and release of ACh requires high amounts of (mitochondrial) energy. Newly released ACh is rapidly broken down by AChE in the synaptic cleft and must be re-synthesized continuously in the cholinergic nerve ending (in contrast to catecholamines or amino acids that can be recycled to a considerable extent [37,38]. What is more, ACh is closely related to learning and memory processes. Cholinergic neurons die early in AD, and this loss contributes strongly to the clinical picture [39,40].

We first noted that ACh levels are considerably lower in transgenic AD rats than in wild-type rats, both in young and aged animals. Judging from the stable ChAT activities, cholinergic cell death does not seem to be prominent in these animals, and a change of AChE activities could also be excluded. In young or old wild-type rats, treatment with nutrients did not affect ACh release. In transgenic rats of either age, however, ACh release was increased by two weeks of treatment with the cocktail when the rats were exposed to the open field (this effect was significant at *p* = 0.044 in old rats and borderline significant at *p* = 0.055 in young rats). These results not only show beneficial effects of a nutrient mixture in an Alzheimer’s model, they also suggest that specific deficits of mitochondrial functions may directly affect the ability of cholinergic neurons to synthesize and release ACh. It seems likely from our data that the ability to synthesize and release ACh is compromised in AD rats, and that this impairment is remedied with a mixture of nutrients that are central to neuronal function. Remarkably, the effect of the cocktail was weaker or not seen at all when scopolamine was used as a pharmacological stimulator. It seems possible, therefore, that the immediate effect of the transgene may be upstream from the septohippocampal pathway and may possibly also involve other neurotransmitter systems, e.g., GABAergic or glutamatergic neurons that also require intact mitochondrial function for maintaining firing rates.

It is speculative what the present results mean for the treatment of human AD. Our data were generated in transgenic rats, and in the worst case, they may only be valid for this specific model bearing two mutations (APP and PS-1) which do not occur in humans simultaneously. Our wild-type and transgenic rats were fed the same diet, but wild-type rats did not respond to the treatment with cocktail although they also had diminished respiration in old age. It follows that the mutations and, possibly, amyloid formation lead to a change of the physiological status of the brain which responds favorably to the ingredients of the cocktail. This is at variance with the conclusion from earlier work in which a multi-nutrient diet had advantageous impacts in old wild-type rats [41].

Our results point to interactions of amyloid peptides with mitochondria in vivo [28]. While the present rat model did not express tau mutations, the TgF-344 rat does develop tau aggregations [12] which are also known to affect mitochondrial function. Tau accumulation interferes with mitochondrial fission and fusion processes, but also with axonal transport and functioning of synaptic mitochondria [42,43], and tau phosphorylation was linked to vitamin B deficiency in tau-transgenic mice [44]. The presence of Aβ may further aggravate tau-induced mitochondrial pathology [45]. While we have no direct evidence of an interference of our treatment approaches on tau function, it is possible that tau aggregation could also be a target of nutrient interventions.

With respect to human patients, it should be noted that AD patients displayed low plasma levels of vitamins and certain minerals in some studies [46]. Furthermore, dietary interventions demonstrated positive effects in patients with late-onset/sporadic AD similar to what we saw in transgenic rats. A combination of nutrients and vitamins was effective in a small case study [47] in which cognitive dysfunction and memory loss could be reversed. However, in this particular study, social interaction and regular exercise were also part of the treatment. In another study, B vitamins, uridine monophosphate, and omega-3 fatty acids improved cognitive function, reduced memory decline, and attenuated disease progression in early stages of AD [48,49]. MRI scans revealed significantly less deterioration in hippocampal volume and in whole brain volume [50]. High doses of folic acid and vitamin B6 and B12 decelerated brain atrophy in patients suffering from mild cognitive impairment [51].

## 4. Materials and Methods

### 4.1. Animals and Treatments

The study was approved and registered by the local authorities (RP Darmstadt; FR1009). All experiments were carried out in accordance with German and European law (EU directive 2010/63/EU) and performed on equal terms with female and male rats. Two age groups were used, 6–7 months or 15–16 months. TgF344-AD rats (RRID: RGD_10401208) and wild type F344 rats (Janvier Labs, Le Geneste St. Isle, France) were bred in controlled rooms (22 °C, 50–65% humidity; day/night cycle of 12/12 h) with access to water and a standard diet (Altromin, Lage, Germany; 1324) ad libitum. In total, 231 animal experiments were conducted, with the rats divided into 6 groups (A–F) with 36–40 animals per group. Animals which were chosen to undergo experiments at the age of 6–7 months (groups A–D) were housed in sets of 4, whereas animals chosen to undergo experiments at the age of 15–16 months (groups E and F) were housed as a pair (two rats per cage). Groups A and E, both controls, received 1 mL of an O/W emulsion (composition shown in Table 2) through oral gavage once a day for 14 days prior to experimentation (B. Braun Injekt^®^ Solo, Melsungen, Germany; 4606108V). Group B received 500 mg/kg magnesium orotate, and group C 300 mg/kg benfotiamine. Groups D and F received a cocktail consisting of magnesium orotate (500 mg/kg), benfotiamine (300 mg/kg), folic acid (10 mg/kg), cyanocobalamin (vitamin B12; 1 mg/kg), and cholecalciferol (vitamin D3; 75 µg/kg) daily for 14 days through oral gavage (all substances supplied by Wörwag Pharma GmbH & Co. KG, Stuttgart, Germany). The time line of the experiments is shown in Figure 1.

The suspensions for gavage were prepared freshly every day and stirred before administration to ensure dosing accuracy. The body weight of male rats at age 6–7 months was 360–390 g, of female rats 220–260 g. 15–16 months old male rats weighed between 480–510 g, whereas female rats did not put on weight with age. During oral gavage the animals’ body weight was daily checked and no loss or gain in weight was noticed, neither were any changes in defecation or behaviour visible.

### 4.2. Mitochondrial Respirometry

After 14 days of oral gavage, 111 rats (6 groups with 18–19 animals) were sacrificed by decapitation in isoflurane anaesthesia. Hippocampi were collected quickly, and mitochondria were isolated. Mitochondrial oxygen consumption was determined in an Oxygraph-2K (O2K) (Oroboros Instruments GmbH, Innsbruck, Austria) [52]. Briefly, the O2K chambers were filled with 2 mL of MiR05 medium (110 mM sucrose, 60 mM K+ lactobionate, 0.5 mM EGTA, 3 mM MgCl2, 20 mM taurine, 10 mM KH2PO4, 20 mM HEPES, 1 g/l fatty acid-free BSA) and 80 μL of isolated mitochondria. Activities of the ETS (electron transfer system) and complexes I, II and IV were determined using an established substrate/uncoupler/inhibitor titration (SUIT) protocol [53,54]. Complex I activity was measured after the addition of pyruvate (5 mM; Sigma-Aldrich, Taufkirchen, Germany, P2256), malate (2 mM; Sigma-Aldrich, Taufkirchen, Germany, M1000), and ADP (2 mM; Sigma-Aldrich, Taufkirchen, Germany, A5285). ATP synthase activity was inhibited by the addition of oligomycin (2 μg/mL; Sigma-Aldrich, Taufkirchen, Germany, O4876). Maximum electron transfer (ETS) was measured after the addition of the protonophore FCCP (Sigma-Aldrich, Taufkirchen, Germany, C2920). Complex II activity was determined after the addition of succinate and inhibition of Complex I by the addition of rotenone (0.5 μM; Sigma-Aldrich, Taufkirchen, Germany, R8875). Then, mitochondrial respiration was blocked by the addition of antimycin-A (Sigma-Aldrich, Taufkirchen, Germany, A8674) which inhibits complex III. Maximum complex IV activity was measured after the addition of tetramethyl-phenylenediamine (TMPD; 0.5 mM; Sigma-Aldrich, Taufkirchen, Germany, A7631) as electron donor and 2 mM ascorbate (Sigma-Aldrich, Taufkirchen, Germany, A7631) to maintain the reduced state of TMPD. Finally, citrate synthase (CS) activity was measured by a colorimetric assay [53]), and oxygen consumption was expressed normalised to CS activities. Normalisation for activity of citrate synthase was used as a quantitative marker of functional mitochondria [55,56].

### 4.3. Microdialysis

Y-shaped concentric microdialysis probes (Polysulfone membrane FX CorDiax 600, Fresenius Medical Care, Bad Homburg, Germany, 0123) with a molecular weight cut-off of 30 kDa and an exchange length of 3 mm were manufactured as described previously [57] and implanted into the lateral hippocampus of fully anaesthetised rats by means of a stereotaxic instrument (Stoelting, Chicago, IL, USA). The coordinates used in the stereotaxic instrument were (from bregma): AP: −5,2 mm; L: −4,8 mm; DV: −7 mm. Isoflurane (Iso-vet, Shanklin, U.K., 3949; induction dose 5%, maintenance dose 1.5–2% v/v) in synthetic air (Air Liquide, 6716684, Düsseldorf, Germany) was used for anaesthesia. Bupivacain (Jenapharm, Jena, Germany) was applied for long-lasting local anaesthesia. In order to confirm the implantation site, the probe was perfused with the dye Fast Green (50 mM in aCSF; Sigma-Aldrich, Taufkirchen, Germany, F7258) and stained brain slices were examined under optical magnification.

After implantation, rats recovered overnight and microdialysis experiments were conducted between 9:00 a.m. and 6:00 p.m. on the two following days, i.e., days 13 and 14 of oral gavage. The probes were perfused with artificial cerebrospinal fluid (aCSF; 147 mM NaCl, 4 mM KCl, 1.2 mM CaCl2, 1.2 mM MgCl2, neostigmine 100 nM) at a rate of 2 µL/min. After 30 min of equilibrium between perfusion liquid and tissue, dialysates were collected in 10 min intervals for 60 min (6 × 20µL) and used for analysis. A total of 120 rats were used for this part of the study (6 groups with 20 animals), but due to blocked probes, 10 rats had to be excluded so that 110 rats were entered into data analysis.

### 4.4. Cholinergic Function

Microdialysis was performed on two consecutive days starting at 09:00 ± 01:00 AM. Artificial cerebrospinal fluid (aCSF) served to perfuse the probes via a microdialysis pump (KD Scientific, Holliston, MA, US) and had the following composition: 147 mM NaCl, 2.7 mM KCl, 1.2 mM MgCl_2_, 1.2 mM CaCl_2_, and 100 nM neostigmine (Acros Organics, Geel, BE). Rats were briefly restrained for connecting the probe to the pump. Prior to sampling, the microdialysate was discarded for 30 min. Then, the perfusion speed was set to 1 μL/min and samples were collected in microvials every 15 min.

As in earlier work [58,59], we used a physiological and a pharmacological stimulus to stimulate ACh release from the septohippocampal pathway. On the first day, basal samples were collected while the animals stayed in their home cages for 90 min. Subsequently, rats were transferred into an open field box (45 × 32 × 20 cm). The animals were able to freely explore the new environment. A maximum of three animals was recorded in parallel in the open field. Open field boxes were cleaned carefully between animals. After 90 min, rats were placed back into their home cages. Sampling was continued for another 90 min to monitor post-interventional ACh release.

On day two, baseline levels were sampled again for 90 min in the home cage. Then, the perfusion fluid was switched to aCSF supplemented with scopolamine (1 μM) for 90 min. Finally, perfusion fluid was switched back to aCSF and samples were again collected for 90 min. After finishing microdialysis on day two, rats were anaesthetised with 5% isoflurane and decapitated. To confirm the implantation site on a random basis, some probes were perfused with the dye Fast Green (50 mM in aCSF; F7258) prior to sacrifice.

For the determination of cholinergic enzyme activities, the brain was harvested directly after decapitation. While kept on an ice-cooled petri dish, the hippocampus was quickly removed and weighed in a cooled potter vessel. Cold HEPES (10 mM)-sucrose (320 mM) buffer (pH 7.4) was added in a ratio of 1:10 (hippocampus: buffer), immediately followed by homogenization (15 hits at 1500 rpm; Potter S, B. Braun, Melsungen, DE). Aliquots of the resulting homogenate were frozen in liquid nitrogen and stored at −80 °C until analysis.

AChE enzyme activity was determined following Ellman’s procedure as detailed previously [60]. The activity of ChAT was determined by the Fonnum procedure which follows the formation of [3H]ACh from [3H]acetyl-Coenzyme A (specific activity: 200 mCi/mmol; Biotrend Chemikalien, Cologne, DE) and choline chloride. Details of the procedure were as published [60]. Protein concentrations were determined by the Bradford procedure using albumin fraction V 96% as standard.

### 4.5. Analytical Measurements

ACh was analysed using high performance liquid chromatography as previously described [56]. Glucose, lactate, and pyruvate levels in dialysates were measured by a colorimetric method using an IscusFlex^®^ microdialysis analyzer (M Dialysis AB, Sweden). The concentration of magnesium in dialysates was measured by atom absorption spectrometry using a PinAAcle 900T (PerkinElmer, Waltham, MA, USA).

### 4.6. Statistical Analysis

Unless otherwise indicated, data are presented as means ± SEM of N (number of animals). All data were tested for normal distribution by the Kolmogorov–Smirnov test (GraphPad Prism 5.03). Potential outliers (>2 SD) were identified by the Grubbs test (https://www.graphpad.com/quickcalcs/grubbs, accessed on 16 September 2021). Sample size was calculated by the formula N = 2 SD2 × power index/delta2. Based on many years of experience, an SD of 20% was expected for ACh measurements and a treatment effect of 25% was defined as goal of the study. The value for the power index (α = 0.05, two-sided; ß = 0.2; 80%) was taken from the book “Intuitive Statistics” by Harvey Motulsky (Oxford University Press, 1995). Treatment effects of four groups on activity changes of mitochondrial complexes (Figure 2A, Figure 3A, Figure 4A and Figure 5A) were compared using one-way analysis of variance (ANOVA; Prism 5.03; GraphPad Software, La Jolla, CA, USA) with Bonferroni’s post-test for multiple pair-wise comparisons. To compare means of genotype, treatment, and age-related effects between two groups on activity changes of mitochondrial complexes, we used the unpaired Student’s *t*-test (Figure 2B, Figure 3B, Figure 4B and Figure 5B). We also used the unpaired Student’s *t*-test to compare means of extracellular magnesium levels between two groups (Figure 6). One-way ANOVA was applied to compare means of six groups on basal values of metabolites (Table 1 and Table 2). Two-way ANOVA was used to compare time courses of ACh measurements in Figure 7, Figure 8, Figure 9, Figure 10, Figure 11 and Figure 12. *p*-values < 0.05 were considered to be statistically significant. All data were normally distributed, and no outliers were detected.

This is an exploratory study using mitochondrial parameters and levels of energy metabolites as major outcome variables. The experimenter was blinded to the animal groups during the measurements of acetylcholine and magnesium. Apart from that, no blinding was performed in this study.

## 5. Conclusions

The TgF344-AD rat model has normal levels of energy substrates in the brain, but it displays distinct mitochondrial dysfunctions not unlike the findings in patients suffering from AD. It should be noted that AD patients have significantly reduced metabolic rates in affected brain regions, but microdialysis data on energy metabolites in AD brain are not available. Reduced mitochondrial respiration found in aged rats was fully restored with a treatment of a “cocktail” in transgenic rats, whereas wild-type rats did not respond. Concomitantly, this cocktail also facilitated the release of hippocampal ACh under during physiological stimulation. The cocktail included magnesium orotate and benfotiamine as well as folic acid, vitamin B12, and vitamin D. In parallel to mitochondrial activities, transgenic rats displayed impairments of cholinergic function that caused reduced levels of hippocampal ACh. When compared to AD patients who have reduced ChAT and AChE activities in the brain, the transgenic rats differ because they have reduced ACh levels but intact enzyme activities and probably no major cholinergic cell loss at 16 months of age. Importantly, however, the cholinergic deficit in transgenic rats that was reflected in lower ACh levels was also attenuated by the nutrient cocktail, possibly because the enhanced mitochondrial function allowed a more prominent ACh synthesis and release. We conclude that the TgF344-AD rat is a promising model of mitochondrial and cholinergic dysfunction. Our results support previous suggestions that nutrient-based therapies may be beneficial for mitochondrial and cholinergic function in AD. 

## Figures and Tables

**Table 1:** Extracellular concentrations of metabolites as determined by microdialysis under basal conditions (not corrected for recovery) in rat hippocampus. Data are means ± SEM (*n* = 8–10) and were analysed by one-way ANOVA. Glucose: F11,109 = 0.57, *p* = 0.85; Lactate: F11,109 = 0.86, *p* = 0.58; Lactate/Pyruvate ratio: F11,109 = 0.70, *p* = 0.73.

Treatment	Glucose [µM]	Lactate [µM]	Lactate/Pyruvate Ratio
Wild type control	157 ± 16	183 ± 19	15 ± 2.5
Transgenic control	180 ± 21	194 ± 8	13.1 ± 0.8
Wt Mg-orotate	161 ± 6	205 ± 19	17.3 ± 2.2
Tg Mg-orotate	152 ± 23	207 ± 23	14 ± 1.6
Wt Benfotiamine	172 ± 11	186 ± 8	15 ± 1.4
Tg Benfotiamine	160 ± 26	163 ± 15	12.7 ± 1.5
Wt Cocktail	167 ± 18	183 ± 18	14.6 ± 0.9
Tg Cocktail	155 ± 18	175 ± 20	14.3 ± 0.7
Old wt control	194 ± 11	210 ± 12	15 ± 0.9
Old tg control	177 ± 10	198 ± 10	14.9 ± 0.7
Old wt Cocktail	174 ± 7	195 ± 10	15.9 ± 1.4
Old tg Cocktail	172 ± 10	197 ± 12	14.1 ± 1.2

**Table 2:** Composition of the emulsion used to administer the cocktail.

Substance	Weighed Portion	% Percentage	Function
Water, deionised	18.75	75	Vehicle
Soy oil	5	20	Vehicle
Glycerol	0.625	2.5	Cosolvent
Lecithin	0.5	2	Emulsifier
Methylcellulose	0.125	0.5	Pseudo-emulsifier

## Data Availability

The data presented in this study are available on request from the corresponding author (patent pending).
